# Lower body mass index and mortality in older adults starting dialysis

**DOI:** 10.1038/s41598-018-30952-2

**Published:** 2018-08-27

**Authors:** Harmke A. Polinder-Bos, Merel van Diepen, Friedo W. Dekker, Ellen K. Hoogeveen, Casper F. M. Franssen, Ron T. Gansevoort, Carlo A. J. M. Gaillard

**Affiliations:** 1 Department of Nephrology, University Medical Center Groningen, University of Groningen, Groningen, The Netherlands; 2 Department of Clinical Epidemiology, Leiden University Medical Center, Leiden, The Netherlands; 3 Department of Nephrology, Jeroen Bosch Hospital, Den Bosch, The Netherlands; 4 Division of Internal Medicine and Dermatology, Department of Nephrology, University Medical Center Utrecht, University of Utrecht, Utrecht, The Netherlands

## Abstract

Lower body mass index (BMI) has consistently been associated with mortality in elderly in the general and chronic disease populations. Remarkably, in older incident dialysis patients no association of BMI with mortality was found. We performed an in-depth analysis and explored possible time-stratified effects of BMI. 908 incident dialysis patients aged ≥65 years of the NECOSAD study were included, and divided into tertiles by baseline BMI (<23.1 (lower), 23.1–26.0 (reference), ≥26.0 (higher) kg/m^2^). Because the hazards changed significantly during follow-up, the effect of BMI was modeled for the short-term (<1 year) and longer-term (≥1 year after dialysis initiation). During follow-up (median 3.8 years) 567 deaths occurred. Lower BMI was associated with higher short-term mortality risk (adjusted-HR 1.63 [1.14–2.32] *P* = 0.007), and lower longer-term mortality risk (adjusted-HR 0.81 [0.63–1.04] *P* = 0.1). Patients with lower BMI who died during the first year had significantly more comorbidity, and worse self-reported physical functioning compared with those who survived the first year. Thus, lower BMI is associated with increased 1-year mortality, but conditional on surviving the first year, lower BMI yielded a similar or lower mortality risk compared with the reference. Those patients with lower BMI, who had limited comorbidity and better physical functioning, had better survival.

## Introduction

Underweight, defined as low body mass index (BMI), has been associated with an excess risk of mortality in older adults^1–9^. Besides in older adults, the deleterious effect of low BMI on survival has been documented in chronic disease populations, including heart failure, chronic obstructive pulmonary disease, peripheral vascular disease, and rheumatoid arthritis^10–14^. Remarkably, a previous study failed to observe an association of BMI with long-term mortality risk in elderly (≥65 years) patients starting dialysis^15^. This finding is striking, as it is inconsistent with the aforementioned effects of low BMI, both in the general older adult population and in chronic disease populations. Moreover, based on clinical experience an absent association of low BMI with survival in older adult dialysis patients seems counterintuitive. Mortality and cardiovascular event rate in dialysis patients are especially high in the first period after start of dialysis, and the effect of risk factors associated with mortality might therefore be different for the short versus longer-term of follow-up^16,17^. Therefore, we conducted an in-depth exploratory analysis of the association of BMI with mortality risk in older incident dialysis patients to investigate whether the effect of baseline-measured BMI on mortality risk might change during follow-up time.

## Results

### Study participants

Of 2,051 patients included in NECOSAD, 925 were aged ≥65 years, and in 908 patients weight and height were available at baseline. Median age of these 908 patients was 73 years (IQR 69–77 years), and median BMI 24.5 kg/m^2^ (IQR 22.5–27.1 kg/m^2^). Three months after dialysis initiation 721 and 176 patients were receiving HD and PD treatment, respectively. Furthermore, 24-hour urinary creatinine excretion indexed by height (UCrE/ht) as a measure of muscle mass, was lower with lower BMI both for men and women. Subjects in the middle and especially the lower BMI tertile had lower muscle mass, as measured by UCrE^18^ (Table 1).

**Table 1:** Baseline Characteristics of older incident dialysis patients according to tertiles of Body Mass Index.

Characteristics	Lower BMI < 23.1 kg/m^2^	Middle BMI 23.1–26.0 kg/m^2^	Higher BMI ≥ 26.0 kg/m^2^
*Total N* = 908	*N* = 302	*N* = 303	*N* = 303
Body mass index (kg/m^2^)	21.5 (20.3–22.3)	24.3 (23.7–25.1)	28.4 (27.1–30.6)
***Demographics***			
Age (yr)	73.1 (69.2–77.4)	73.1 (69.0–76.9)	72.3 (69.1–76.5)
Men (%)	195 (65)	196 (65)	172 (57)
Non-Caucasian race (%)	18 (6)	17 (6)	14 (5)
Primary kidney disease (%)			
Glomerulonephritis	17 (6)	24 (8)	20 (7)
Diabetes mellitus	24 (8)	35 (12)	71 (23)
Renal vascular disease	83 (28)	92 (30)	66 (22)
Other	178 (59)	152 (50)	146 (48)
Educational level (%) (*N* = 737)			
Level 1	8 (3)	9 (4)	6 (2)
Level 2	19 (8)	18 (7)	14 (6)
Level 3	24 (10)	29 (12)	27 (11)
Level 4	91 (39)	99 (40)	96 (38)
Level 5	92 (39)	92 (37)	113 (44)
Current smoking (%) (*N* = 797)	75 (29)	51 (19)	35 (13)
Systolic blood pressure (mmHg)	148 ± 25	153 ± 35	151 ± 23
Diastolic blood pressure (mmHg)	79 (70–85)	80 (70–86)	80 (70–86)
Mean arterial pressure (mmHg)	101 ± 14	104 ± 15	102 ± 14
*Comorbidities* (%) (*N* = *800*)			
Myocardial infarction	51 (19)	63 (23)	45 (17)
Heart failure	59 (22)	60 (22)	41 (16)
Diabetes mellitus	45 (17)	62 (23)	103 (39)
Peripheral vascular disease	65 (25)	60 (22)	62 (24)
Cerebrovascular accident	32 (12)	28 (10)	35 (13)
Malignancy	49 (19)	42 (15)	34 (13)
Chronic lung disease	29 (11)	31 (11)	29 (11)
Chronic infection	6 (2)	7 (2)	3 (1)
*Self-reported physical functioning*			
EQ. 5D mobility (%) (*N* = 724)			
no limitations in walking	62 (27)	58 (24)	48 (19)
some limitations in walking	154 (67)	174 (72)	195 (77)
confined to bed	13 (6)	10 (4)	10 (4)
EQ. 5D usual activities (%) (UA) (*N* = 721)			
no limitations in performing UA	52 (23)	63 (26)	39 (15)
some limitations in performing UA	112 (49)	112 (47)	132 (52)
not able to perform UA	65 (28)	64 (27)	82 (32)
***Laboratory results***			
eGFR (ml/min) (*N* = 699)	7.5 (5.7–9.5)	7.5 (5.9–9.7)	7.6 (6.1–9.3)
Albumin (g/L) (*N* = 829)	33.7 ± 5.9	35.3 ± 5.6	35.1 ± 6.0
Urea (mmol/L) (*N* = 864)	33.7 ± 11.8	31.1 ± 10.4	31.4 ± 9.9
UCrE/height (mmol/24 h/m)			
Men (*N* = 267)	4.2 ± 1.3	4.5 ± 1.2	5.0 ± 1.4
Women (*N* = 171)	3.3 ± 0.9	3.7 ± 1.2	3.8 ± 1.1
Low UCrE (%) (*N* = 438) *	81 (55)	59 (44)	56 (36)

Data are expressed as mean ± SD or median (interquartile range). Educational level 1 = university. *Low muscle mass was defined using a previously developed regression formula^18^. Abbreviations; eGFR, estimated glomerular filtration rate; EQ. 5D, EuroQol 5-dimensional questionnaire; MDRD, modification of diet in renal disease; UA, usual activities; UCrE, 24-hour urinary creatinine excretion.

### BMI and mortality risk

**Figure 1:**
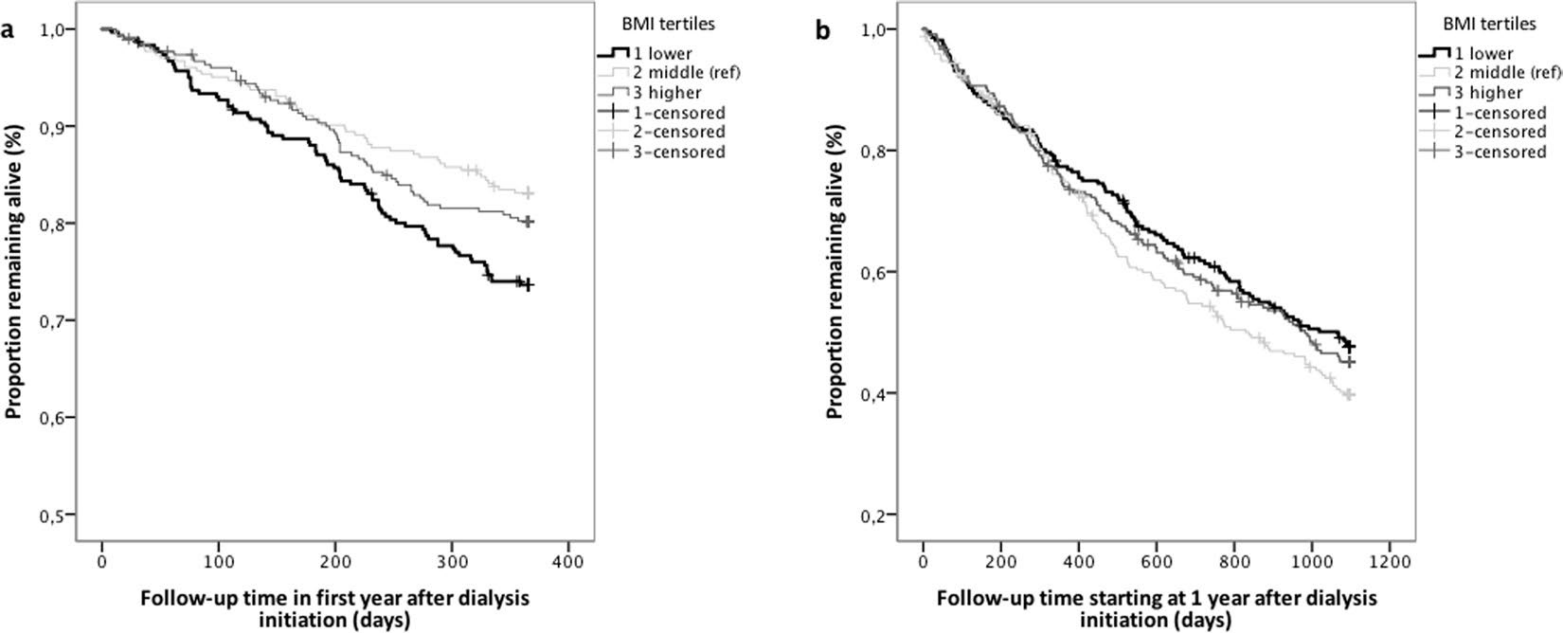
Kaplan Meier survival curves for all-cause mortality (**a**) in the first year of dialysis, and (**b**) thereafter. (**a** and **b**) Kaplan Meier survival curves for all-cause mortality by Body Mass Index (BMI) tertiles in 908 elderly participants of the NECOSAD study. Upper panel (1a): Follow up during the 1st year after dialysis initiation. Numbers at risk were: 908 (lower BMI: 302, middle BMI: 303; higher BMI: 303). Log rank test: χ^2^(2) = 8.409, P = 0.01. Lower panel (1b): Follow up after the 1st year of dialysis treatment. Numbers at risk were: 701 (lower BMI: 217; middle BMI: 248; higher BMI: 236). Log rank test: χ^2^(2) = 2.875, P = 0.2.

During follow-up 567 patients died, 43 patients were censored because they received a kidney transplantation, 16 because of recovery of kidney function, and 6 because of loss to follow-up. Mean follow-up time was 3.8 years. The cumulative survival proportion at the end follow-up was: 30%, 28% and 31% for the BMI tertiles 1–3, respectively (log-rank test, *P* = 0.8). Supplementary Fig. S1 shows the Kaplan Meier curves with non-proportional hazards during follow-up. The subdivided Kaplan Meier curves for short-term and longer-term follow-up indicated that lower BMI patients had a higher mortality risk during the first year after dialysis initiation, whereas mortality risk was similar thereafter (Fig. 1). This time-stratified effect of lower BMI was confirmed by a significant global PH assumption test (*P* = 0.03), with the detailed test indicating violation of the PH assumption by lower BMI (*P* = 0.01). Similarly, adding an interaction term of lower BMI with time to the Cox regression model yielded a significant interaction (*P* = 0.01). Therefore, we subsequently analyzed mortality risk for the first year, and for the years thereafter separately.

**Figure 2:**
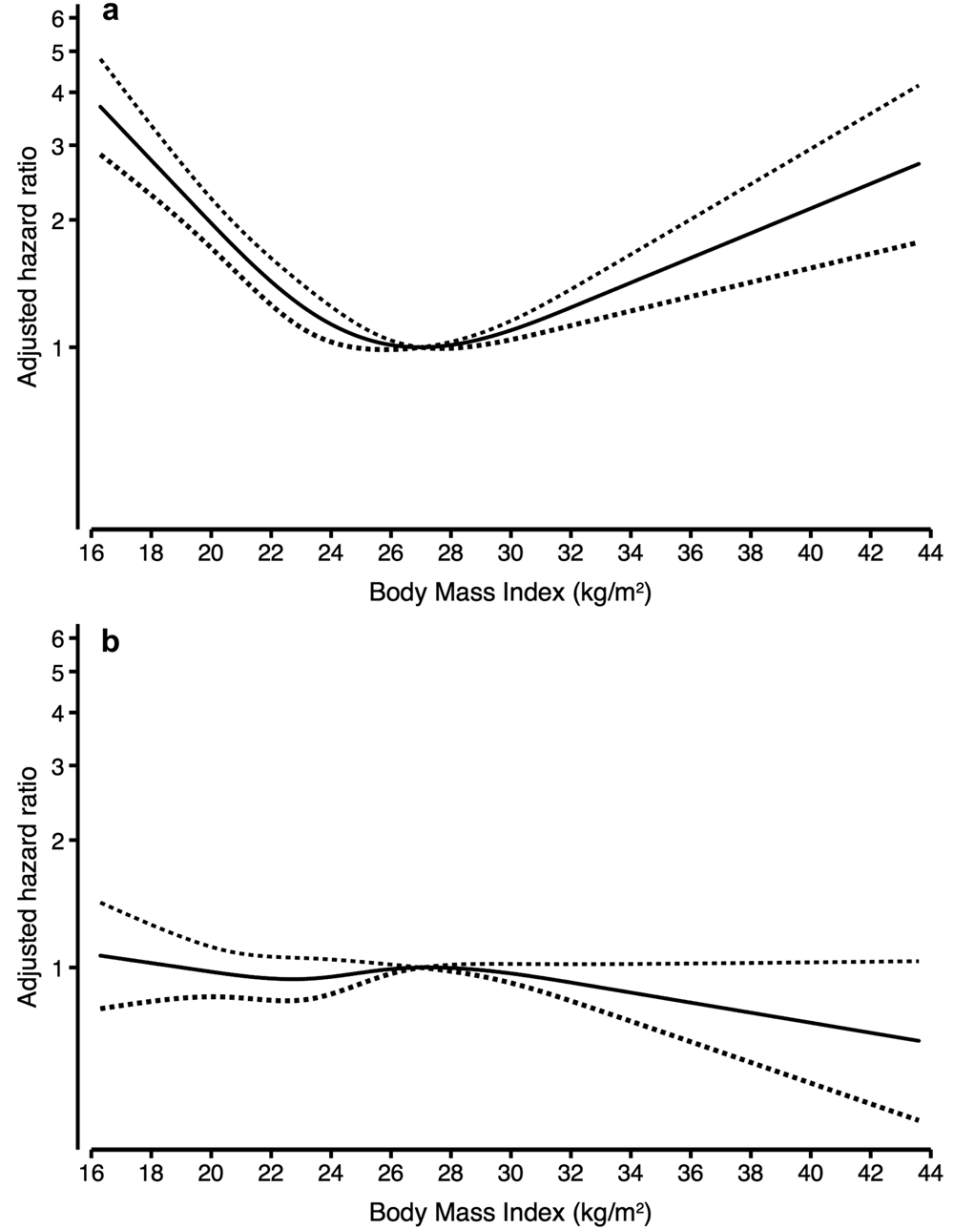
Relation between Body Mass Index and mortality during (**a**) the first year of dialysis, and (**b**) thereafter. Hazard ratios for mortality depending on body mass index (BMI) were modeled by a restricted cubic spline in a Cox regression model. The model was adjusted for age, sex, smoking, and race. The upper panel (a) shows the mortality risk during 1st year after dialysis initiation, whereas the lower panel (b) shows the mortality risk thereafter.

Visualizing the effect of BMI on mortality risk during these two time periods using restricted cubic splines, clearly showed a different pattern for mortality risk for lower BMI values during the first year of dialysis when compared to the years thereafter (Fig. 2). Lower BMI (<23.1 kg/m^2^) was associated with a higher mortality risk in the first year of dialysis initiation both crude (HR 1.64 [1.16–2.34] *P* = 0.006) and adjusted for age, sex, race, primary kidney disease, and smoking (HR 1.63 [1.14–2.32] *P* = 0.007) (Table 2). Conditional on surviving the first year of dialysis therapy, lower BMI yielded a similar mortality risk compared with normal BMI, both crude (HR 0.81 [0.63–1.04] *P* = 0.1), and adjusted for age, sex, race, primary kidney disease, and smoking (HR 0.81 [0.63–1.04] *P* = 0.1) (Table 2). Further adjusting for comorbidities, SBP, albumin and dialysis modality slightly modified the hazard ratios (HR) for the first year (HR 1.49 [1.04–2.13] *P* = 0.03), and thereafter (HR 0.77 [0.60–0.99] *P* = 0.04). Higher BMI yielded a similar mortality risk compared with the middle BMI tertile.

**Table 2:** Body Mass Index and all-cause mortality risk in the first year of dialysis and thereafter.

BMI tertiles	Number of events/pts	Hazard ratio (95% Confidence Interval)		
		Model 1	Model 2	Model 3
***The First Year of Dialysis*** **:**				
<23.1 kg/m^2^	79/302	1.64 (1.16–2.34)**	1.63 (1.14–2.32)**	1.49 (1.04–2.13)*
23.1–26.0 kg/m^2^	51/303	1.00 (ref)	1.00 (ref)	1.00 (ref)
≥26.0 kg/m^2^	59/303	1.19 (0.82–1.73)	1.17 (0.80–1.70)	1.10 (0.76–1.61)
***After the First Year of Dialysis*** **:**				
<23.1 kg/m^2^	111/217	0.81 (0.63–1.04)	0.81 (0.63–1.04)	0.77 (0.60–0.99)*
23.1–26.0 kg/m^2^	142/248	1.00 (ref)	1.00 (ref)	1.00 (ref)
≥26.0 kg/m^2^	125/236	0.87 (0.69–1.11)	0.88 (0.69–1.11)	0.83 (0.65–1.06)

Model 1 = crude; Model 2 = adjusted for age, gender, race, primary kidney disease, and smoking; Model 3 = model 2+ albumin, systolic blood pressure, comorbidities, and treatment modality. *P < 0.05, **P < 0.01.

Causes of death were recorded and known in 410 of 567 deaths (72%). The higher mortality risk in the first year of dialysis in lower BMI patients was especially caused by non-cardiovascular death (Supplementary Table S1A), whereas the favorable effect of lower BMI on mortality risk after the first year of dialysis seemed driven by a lower cardiovascular mortality risk (Supplementary Table S1B).

### Sensitivity analyses

Including survival time and death after kidney transplantation yielded one more death (568) and did not essentially change our results (Supplementary Table S2). Similarly, not adjusting for diabetes mellitus, systolic blood pressure and primary kidney disease yielded similar results for lower BMI in the first year of follow-up (HR 1.49 [1.04–2.12], *P* = 0.03), and thereafter (HR 0.75 [0.58–0.96], *P* = 0.02).

Categorizing BMI according the WHO guidelines resulted in 61 patients having underweight, 458 patients with normal weight, 299 patients with overweight, and 90 patients with obesity. Underweight patients (BMI < 20 kg/m^2^) had a higher mortality risk that was similar to the BMI-tertiles analysis, although the association did not reach formal statistical significance due to the smaller size of this subgroup (HR 1.61 [1.00–2.61] *P* = 0.053, adjusted for age, sex, race, primary kidney disease, and smoking). Conditional on having survived the first year of dialysis therapy, underweight yielded a similar mortality risk as compared with patients with a normal weight (HR 1.04 [0.64–1.70] *P* = 0.9). Finally, dialysis modality did not interact significantly with BMI tertile.

### Post-hoc analysis of lower BMI patients who died during the first year versus those who survived the first year

Of the lower baseline BMI tertile (302 patients), 79 died and 6 were censored (2 for kidney transplantation, 3 for recovery of renal function, and 1 for loss to follow-up) during the first year after dialysis initiation. Comorbidity burden was significantly higher among these 79 non-survivors as compared with the patients who survived the first year of dialysis therapy (Table 3). Furthermore, the non-survivors had a significantly worse self-reported physical functioning. A multivariable logistic regression model showed that in lower BMI patients the presence of peripheral vascular disease, heart failure, diabetes mellitus, and malignancy, and higher urea and lower albumin blood levels were risk factors associated with dying in the first year (Supplementary Table S3).

**Table 3:** Characteristics of lower Body Mass Index patients (<23.1 kg/m^2^) not surviving versus surviving the first year of dialysis, measured at baseline.

Characteristics *Total N* = 302	Death <1 year after start of dialysis *N* = 79	Surviving year 1 of dialysis *N* = 222	*P*
***Demographics***			
Age	74.1 (70.5–79.0)	72.9 (68.8–77.2)	0.1
Men (%)	55 (70)	139 (63)	0.3
Non-Caucasian race (%)	5 (6)	13 (6)	1.0
Educational level (%) (*N* = 233)			0.6
Level 1	0 (0)	8 (4)	
Level 2	5 (10)	14 (8)	
Level 3	5 (10)	19 (10)	
Level 4	19 (38)	71 (39)	
Level 5	21 (42)	71 (39)	
Primary kidney disease (%)			0.006
Glomerulonephritis	2 (3)	15 (7)	
Diabetes mellitus	12 (15)	12 (5)	
Renal vascular disease	27 (34)	56 (25)	
Other	38 (48)	139 (63)	
Body mass index (kg/m^2^)	20.9 ± 1.7	21.2 ± 1.5	0.2
Current smoking (*N* = 263)	23 (33)	52 (27)	0.4
Systolic blood pressure	145 ± 28	149 ± 24	0.2
Diastolic blood pressure	75 (65–80)	80 (70–85)	0.06
Mean arterial pressure	98 ± 15	102 ± 14	0.07
Hemodialysis 3 months after start (*N* = 298)	67 (86)	181 (82)	0.6
***Comorbidities*** (**%**) (***N*** = ***263***)			
Myocardial infarction	19 (26)	32 (17)	0.08
Heart failure	26 (36)	33 (17)	0.001
Diabetes mellitus	20 (28)	25 (13)	0.005
Peripheral vascular disease	30 (42)	35 (18)	<0.001
Cerebrovascular accident	12 (17)	20 (11)	0.2
Malignancy	20 (28)	29 (15)	0.02
Chronic lung disease	14 (19)	15 (8)	0.007
Chronic infection	1 (1)	5 (2)	1.0
***Self-reported physical functioning*** **(*N*** = ***228*)**			
EQ. 5D mobility			0.002
no limitations in walking	5 (10)	57 (32)	
some limitations in walking	37 (77)	116 (64)	
confined to bed	6 (13)	7 (4)	
EQ. 5D usual activities (UA)			0.005
no limitations in performing UA	5 (10)	47 (26)	
some limitations in performing UA	21 (44)	90 (50)	
not able to perform UA	22 (46)	43 (24)	
***Laboratory results***			
eGFR MDRD (*N* = 226)	8.2 (6.0–10.4)	7.3 (5.7–9.3)	0.2
Albumin (g/L) (*N* = 280)	32.2 ± 5.9	34.2 ± 5.8	0.009
Urea (mmol/L) (*N* = 291)	36.1 ± 12.7	32.8 ± 11.4	0.03
UCrE/height (mmol/24 h/m)			
Men (*N* = 89)	3.8 ± 1.1	4.4 ± 1.3	0.07
Women (*N* = 58)	3.1 ± 1.0	3.3 ± 0.9	0.4
Low UCrE* (*N* = 146)	29 (66)	51 (50)	0.08

Data are expressed as mean ± SD or median (interquartile range). Educational level 1 = university. *Low muscle mass was defined using a previously developed regression formula^18^. Abbreviations: eGFR, estimated glomerular filtration rate; EQ. 5D, EuroQol 5-dimensional questionnaire; MDRD, modification of diet in renal disease equation; UCrE, 24-hour urinary creatinine excretion.

## Discussion

Previously, among older incident dialysis patients no association of BMI with long-term mortality risk was reported^15^. Since this finding is at variance with findings in the literature in other cohorts, we explored the association of BMI and mortality in more detail. The main finding of this study was a time-stratified association of BMI with mortality. Indeed, for the long-term follow up no association of BMI with survival was found, and the BMI tertiles yielded equal cumulative survival percentages. However, at the short-term, lower BMI was associated with a significantly higher mortality risk during the first year of dialysis therapy. Thereby, this study adds to previous findings on the association of BMI with mortality in incident dialysis patients^15,19^.

The higher mortality risk for older adults who initiate dialysis with lower BMI is in keeping with another study. Calabia *et al*. reported highest mortality risks for those with lower BMI levels (BMI < 23 kg/m^2^) with an essentially similar risk for those aged <65 years versus ≥65 years, during a median follow-up of 3.6 years, although they did not adjust for the confounding effect of smoking^20^. Interestingly, the authors mentioned a non-proportional risk over time for BMI and used a parametric model, but the actual change in mortality risk over time was not reported. Three other studies that evaluated BMI in older dialysis patients included only prevalent, or both incident and prevalent dialysis patients without reporting the separate results for older adult incident dialysis patients, thus rendering comparison with our results inappropriate^21–23^.

A first question that arises in this study is why lower BMI is associated with such a marked short-term excess mortality. First, lower BMI might be a consequence of inflammation and protein energy wasting (PEW) in CKD patients^24–26^, both conditions that are associated with mortality. In a recent study among hemodialysis patients, low BMI yielded a markedly higher mortality risk in inflamed patients compared with non-inflamed patients^27^. Lower BMI as part of the PEW/ inflammation- complex might especially result in a high mortality in the short-term. The findings of our study support this hypothesis, because the lower BMI patients that did survive the first year had a lower comorbidity burden and higher albumin levels at baseline suggesting less inflammation. Of note, between BMI groups the comorbidity burden was surprisingly similar except for diabetes mellitus that showed a higher prevalence in higher BMI patients as expected. Second, alternatively low BMI could be the result of low food intake, due to poor appetite and dietary restrictions, which are both also associated with worse outcomes^28–30^. Third, patients with low BMI are more likely to have a low muscle mass, which associates with high mortality rates^31–34^.

A remarkable aspect of our study is the observed time-stratified association of lower baseline BMI with mortality that has not been reported previously in older incident dialysis patients. This raises a second question why lower BMI yields a higher mortality risk in the first year of dialysis therapy only in our cohort. In our view, it is understandable that the associated effect of BMI, measured at baseline, is highest early in follow-up in incident patients, as with longer follow-up duration, especially during the first year of dialysis therapy, many changes in weight and health might occur^35,36^. Besides, other studies have shown that changes, especially decreases, in BMI over a relatively short period of time are associated with higher risk of subsequent hospitalization^37^, and with a higher mortality risk^38,39^.

The present study has several strengths. First, as this cohort included only incident dialysis patients the issue of survivor bias does not apply for the first year of follow-up. Second, the issue of immortal time bias also does not apply to this study, as all patients started dialysis at baseline and we used time-stratified Cox-models thereby modeling the whole follow-up period. Third, because the NECOSAD cohort is a well-phenotyped cohort, we were able to correct for important confounders like smoking and albumin, as a measure of inflammation.

Some limitations need to be considered. First, we categorized BMI in tertiles instead of using the thresholds as defined by the WHO guidelines. This was done to allow more power, because the use of the WHO thresholds resulted in some very small subgroups. In addition, a BMI < 23 kg/m^2^ has been recommended as threshold to screen for PEW together with other nutritional markers in the CKD population^40,41^. Second, the baseline BMI measurement before dialysis initiation might have been influenced by fluid overload leading to a higher BMI. This would increase all BMI values yielding more patients in especially the higher BMI tertile, but it is unlikely that this would have changed our findings for lower BMI. Third, we used a mainly Caucasian population limiting generalizability. At the same time, this might have prevented potential complicating factors including race-specific cut-off points for low BMI^42^. Fourth, BMI does not distinguish between tissues and includes both fat and muscle mass. Therefore, lower BMI might imply a low fat mass, a low muscle mass, or both. The lack of further data on body composition at baseline limits a more detailed interpretation of the effect of lower BMI (i.e. the relative contribution of lean and adipose tissue) on mortality risk. Nevertheless, muscle function might be more important than muscle mass in the analysis of mortality risk^33^, and additionally adjusting for physical functioning did not essentially change our findings (results not shown). Furthermore, it should be noted that the inclusion of the NECOSAD population was started in 1997. Although standard of care of dialysis treatment in the Netherlands was already high at that time, it cannot be excluded that NECOSAD is not fully representative for a contemporary dialysis population. However, it is unlikely that the time-stratified effect of lower BMI on mortality risk in older incident dialysis patients will be affected by the small changes in treatment during the past decade. Finally, because we used baseline data, we ware not able to take information on treatment adequacy (i.e. Kt/V) into account.

Older adults with CKD progressing to end-stage renal disease face the dilemma whether to start renal replacement therapy, or choose supportive management. Because in this group the outcome after initiation of renal replacement therapy is highly heterogeneous, various prediction scores have been developed in order to help clinicians and patients in the decision-making process and included BMI^43,44^. Although we did not perform a prediction study, our results seem to indicate that besides lower BMI, mobility, and comorbidities including diabetes mellitus, heart failure, malignancy, and peripheral vascular disease, and albumin and urea levels should be included in prediction scores of short-term mortality as well. Therapeutic decisions such as not initiating dialysis therapy however cannot be based on this study, since we performed a post-hoc analysis and thus confounding cannot be excluded.

In conclusion, in older incident dialysis patients lower baseline BMI is associated with increased 1-year mortality. Remarkably, conditional on surviving the first year of dialysis, patients with lower BMI had a similar or even lower mortality risk compared with the reference BMI. Those patients with lower BMI, who had limited comorbidity and better physical functioning, had better survival.

## Materials and Methods

### Study Design and Population

For the present study data of the NECOSAD study were used. NECOSAD is an observational, prospective cohort study in which 2051 consecutive incident dialysis patients were enrolled between January 1997 and April 2004 in 38 participating dialysis centers across the Netherlands. Patients were followed from study inclusion at the start of dialysis until the end of follow-up on May 26 2009, kidney transplantation, death or until loss to follow-up. The institutional review board of the Academic Medical Hospital, Amsterdam, the Netherlands approved the study, and the institutional review boards of all participating hospitals confirmed this by an additional local approval. All patients gave written informed consent. The study was performed in accordance with the relevant guidelines and regulations. Detailed information on the design of the NECOSAD study has been published previously^45^. For this present study, we included participants who were aged ≥65 years at baseline, in whom height and weight were available. We focused on early mortality and therefore censored follow-up at four years after dialysis initiation.

### Demographic and clinical data

All demographic and clinical data were collected at start of dialysis treatment. Primary kidney disease was classified according to the codes of the European Renal Association–European Dialysis and Transplant Association (ERA-EDTA)^46^. Patients were grouped into four classes of primary kidney disease: glomerulonephritis, diabetes mellitus, renal vascular disease, and other kidney diseases. Educational levels were categorized according to the International Standard Classification of Education as primary or below primary education (level 1), lower secondary education (level 2), upper secondary education (level 3), postsecondary, nontertiary or short-cycle tertiary education (level 4), bachelor master or doctorate graduate (level 5)^47^. Current smokers included those who quitted smoking within the past 3 months. The subscales mobility and usual activities according the EuroQol 5-dimensional (EQ-5D) questionnaire were used as proxies of self-reported physical functioning^48^. Glomerular filtration rate (GFR) was calculated as the mean of urea and creatinine clearance, measured from 24-hour urine collections. The abbreviated modification of diet in renal disease equation was used to estimate GFR (eGFR). 24-hour urinary creatinine excretion (UCrE), as a measure for muscle mass, has previously been defined as low or normal, according the sex- and age-specific 5^th^ percentile of a healthy population^18^.

### Outcome

The main outcome of this study was all-cause mortality. For our analyses, we assessed time to death from the date of dialysis initiation onwards. Patient survival was censored at kidney transplantation, loss to follow-up, or at the end of follow-up. For the cause-specific analyses, cardiovascular mortality was defined as death attributable to myocardial ischemia and infarction, heart failure, cardiac arrest because of other or unknown cause, or cerebrovascular accident (ERA-EDTA codes 11, 14–16, 18, and 22). Noncardiovascular mortality was defined as all other known causes of death.

### Statistical analyses

Variables are presented as mean ± SD, median (interquartile range), or number (percentage) where appropriate. Patients were stratified according baseline BMI tertiles, measured at start of dialysis. We used baseline BMI instead of repeated BMI measurements, because we were interested in the effect of pre-dialysis BMI rather than changes in BMI on mortality risk.

Information on smoking, albumin, comorbidities, and dialysis modality was missing in 1–12% of cases, and missing values were imputed by multiple imputation using 10 repetitions.

We used life tables to calculate cumulative proportions surviving during follow-up. Kaplan Meier curves were used to study the hazards for every BMI tertile. The proportional hazard (PH) assumption was tested using Schoenfeld residuals.

Time-stratified Cox-regression analyses were performed to estimate the time-stratified hazard ratios (HR) and 95% confidence intervals for the mortality risks associated with BMI tertiles. Sequential models were developed to correct for different sets of confounding factors and are presented crude (model 1), and subsequently also adjusted for age, gender, race, primary kidney disease, and smoking (model 2), and additionally for albumin, systolic blood pressure, treatment modality at 3 months follow-up, and comorbidities (history of cardiovascular disease, malignancy, chronic lung disease, diabetes mellitus) (model 3). The middle BMI tertile was used as the reference. Because the hazard changed significantly over time, follow-up time was divided in the first year of follow up to model short-term mortality, and the follow-up thereafter, i.e. longer-term mortality. For the time-stratified Cox-regression analysis, we added two BMI time-stratified covariates (time <366 days * BMI tertile; time ≥366 days * BMI tertile) to the model. The different association of BMI with mortality risk according length of follow up was visualized by four-knot restricted cubic splines for the short-term and longer-term follow up separately. The knots were chosen at the 5th, 35th, 65th, and 95th percentiles of the BMI distribution.

Several sensitivity analyses were performed. First, the time-stratified Cox-regression analyses were repeated without censoring for kidney transplantation, and follow-up time was extended to death after transplantation or censoring at 4 years of follow-up. Information on survival after kidney transplantation was available from the Dutch national renal data system (Renine). Secondly, the time-stratified Cox-regression analyses were repeated without adjustment for diabetes mellitus, systolic blood pressure and primary kidney disease, because those factors might be in the causal pathway between BMI and death. Thirdly, BMI was analyzed in categories according to the World Health Organization (WHO) guidelines in underweight (BMI < 20 kg/m^2^), normal weight (BMI 20–25 kg/m^2^), overweight (BMI ≥ 25 kg/m^2^) or obesity (BMI ≥ 30), with normal weight as the reference category. Finally, we tested whether there was effect modification of dialysis modality by including an interaction term with BMI.

In a post-hoc analysis, we explored characteristics of lower BMI patients who survived the first year compared with those who died, in order to identify correlates for future prognostic studies that potentially might have favored survival. Differences were tested for statistical significance using the Chi-square test, the T-test or Mann-Whitney test, as appropriate. In addition, we used logistic regression modeling (univariable and multivariable) to study potential risk factors for dying in the first year of dialysis treatment in lower BMI patients.

A *P* value < 0.05 was considered statistically significant. The analyses were performed in SPSS version 23.0 (SPSS Inc, IBM company, USA), and in Stata/Se 14.2 (StataCorp LLC, USA).

## Electronic supplementary material

Supplementary material

## Data Availability

All data generated or analyzed during this study are included in this published article. The datasets used and/or analyzed during the current study are available (conditional on agreement on privacy matters and appropriate usage of the data) upon request from the Department of Clinical Epidemiology of the Leiden University Medical Center (Datamanager: Ingeborg de Jonge, Data management office, Department of Clinical Epidemiology C7-P, Leiden University Medical Center, P.O. Box 9600, 2300 RC Leiden, The Netherlands, email: i.de_jonge@lumc.nl).
