# Publisher Correction: Lower body mass index and mortality in older adults starting dialysis

**DOI:** 10.1038/s41598-018-32555-3

**Published:** 2018-09-18

**Authors:** Harmke A. Polinder-Bos, Merel van Diepen, Friedo W. Dekker, Ellen K. Hoogeveen, Casper F. M. Franssen, Ron T. Gansevoort, Carlo A. J. M. Gaillard

**Affiliations:** 1 Department of Nephrology, University Medical Center Groningen, University of Groningen, Groningen, The Netherlands; 2 Department of Clinical Epidemiology, Leiden University Medical Center, Leiden, The Netherlands; 3 Department of Nephrology, Jeroen Bosch Hospital, Den Bosch, The Netherlands; 4 Division of Internal Medicine and Dermatology, Department of Nephrology, University Medical Center Utrecht, University of Utrecht, Utrecht, The Netherlands

Correction to: *Scientific Reports* 10.1038/s41598-018-30952-2, published online 27 August 2018

I. de Jonge did not contribute directly to this study and has been removed from the author list. Correspondence and requests for materials should be addressed to H.A.P.-B. (email: H.A.Polinder-Bos@umcg.nl).

This error has now been corrected in the PDF and HTML versions of the Article.

