# Supplementary material for: Lower body mass index and mortality in older adults starting dialysis
Source: Sci Rep. 2018 Aug 27;8:12858. doi: 10.1038/s41598-018-30952-2 (PMC6110755; doi:10.1038/s41598-018-30952-2)
Supplement: Supplementary file 1 — Supplementary material [file 41598_2018_30952_MOESM1_ESM.docx]

**Supplementary file to manuscript:**

“Lower body mass index and mortality in older adults starting dialysis”

Harmke A. Polinder-Bos MD, Merel van Diepen PhD, Friedo W. Dekker MD PhD, Ellen K. Hoogeveen MD PhD, Casper F.M. Franssen MD PhD, Ron T. Gansevoort MD PhD and Carlo A.J.M Gaillard MD PhD

**Including:**

**Table S1** Body Mass Index and cause-specific mortality risk in the first year of dialysis, and thereafter.

**Table S2** Body Mass Index and mortality risk in the first year of dialysis, and thereafter. Without censoring for kidney transplantation.

**Table S3** Logistic regression model of variables associated with dying in the first year of dialysis treatment versus surviving, in lower Body Mass Index patients (<23.1 kg/m^2^) measured at baseline.

**Figure S1** Kaplan Meier survival curves for all-cause mortality by Body Mass Index tertiles.

**Table S1** Body Mass Index and cause-specific mortality risk in the first year of dialysis (panel A), and thereafter (panel B).

|  |  | Hazard ratio (95% Confidence Interval) | | | | |
| --- | --- | --- | --- | --- | --- | --- |
| BMI tertiles | **Number of events/pts** | **Model 1** | | **Model 2** | **Model 3** | |
|  | |  | |  |  | |
| *Panel A*  *During the first year of dialysis:* | |  | |  |  | |
| *Cardiovascular death:* | |  | |  |  | |
| < 23.1 kg/m^2^ | 19/292 | 1.50 (0.74-3.03) | | 1.52 (0.75-3.09) | 1.46 (0.71-2.97) | |
| 23.1 - 26.0 kg/m^2^ | 13/293 | 1.00 (ref) | | 1.00 (ref) | 1.00 (ref) | |
| ≥ 26.0 kg/m^2^ | 20/293 | 1.57 (0.78-3.15) | | 1.63 (0.81-3.29) | 1.58 (0.78-3.20) | |
| *Non-cardiovascular death:* | |  | |  |  | |
| < 23.1 kg/m^2^ | 50/292 | 1.86 (1.17-2.95)** | | 1.83 (1.15-2.92)* | 1.61 (1.01-2.58)* | |
| 23.1 - 26.0 kg/m^2^ | 28/293 | 1.00 (ref) | | 1.00 (ref) | 1.00 (ref) | |
| ≥ 26.0 kg/m^2^ | 29/293 | 1.07 (0.64-1.79) | | 1.04 (0.61-1.75) | 0.96 (0.57-1.63) | |
|  |  |  | |  |  | |
|  | | | |  |  | |
| *Panel B*  *After the first year of dialysis:* | | | |  |  | |
| *Cardiovascular death* | | |  |  |  |  |
| < 23.1 kg/m^2^ | 21/178 | | 0.56 (0.33-0.96) | 0.59 (0.34-1.00) | 0.57 (0.33-0.97)* |  |
| 23.1 - 26.0 kg/m^2^ | 39/202 | | 1.00 (ref) | 1.00 (ref) | 1.00 (ref) |  |
| ≥ 26.0 kg/m^2^ | 40/194 | | 1.00 (0.65-1.56) | 1.03 (0.66-1.62) | 1.00 (0.64-1.57) |  |
| *Non-cardiovascular death* | | |  |  |  |  |
| < 23.1 kg/m^2^ | 51/178 | | 0.94 (0.64-1.37) | 0.93 (0.64-1.37) | 0.88 (0.60-1.29) |  |
| 23.1 - 26.0 kg/m^2^ | 57/202 | | 1.00 (ref) | 1.00 (ref) | 1.00 (ref) |  |
| ≥ 26.0 kg/m^2^ | 43/194 | | 0.74 (0.50-1.10) | 0.75 (0.50-1.12) | 0.71 (0.48-1.07) |  |
| *The analysis was performed in a subgroup of 410 patients. Model 1= crude; Model 2= adjusted for age, gender, race, primary kidney disease, and smoking; Model 3= model 2 + albumin, SBP, comorbidities, and treatment modality. * P<0.05, ** P<0.01.* | | | | | | |

**Table S2** Body Mass Index and mortality risk in the first year of dialysis (Panel A), and thereafter (Panel B). Without censoring for kidney transplantation.

|  | |  | | Hazard ratio (95% Confidence Interval) | | |
| --- | --- | --- | --- | --- | --- | --- |
| BMI tertiles | | **Number of events/pts** | | **Model 1** | **Model 2** | **Model 3** |
|  | | | |  |  |  |
| *Panel A*  *During the first year of dialysis:* | | | |  |  |  |
| < 23.1 kg/m^2^ | | 79/302 | | 1.64 (1.16-2.34)** | 1.67 (1.17-2.37)** | 1.49 (1.04-2.12)* |
| 23.1 - 26.0 kg/m^2^ | | 51/303 | | 1.00 (ref) | 1.00 (ref) | 1.00 (ref) |
| ≥ 26.0 kg/m^2^ | | 58/303 | | 1.17 (0.80-1.71) | 1.12 (0.77-1.64) | 1.08 (0.74- 1.58) |
|  | |  | |  |  |  |
| *Panel B*  *After the first year of dialysis:* | | | |  |  |  |
| < 23.1 kg/m^2^ | 111/217 | | 0.85 (0.66-1.09) | | 0.85 (0.66-1.09) | 0.78 (0.61-1.01) |
| 23.1 - 26.0 kg/m^2^ | 141/248 | | 1.00 (ref) | | 1.00 (ref) | 1.00 (ref) |
| ≥ 26.0 kg/m^2^ | 127/236 | | 0.92 (0.73-1.17) | | 0.89 (0.70-1.14) | 0.86 (0.67-1.10) |
| *Model 1= crude; Model 2= adjusted for age, gender, race, primary kidney disease, and smoking; Model 3= model 2 + albumin, SBP, comorbidities, and treatment modality. * p<0.05, ** p<0.01, *** p<0.001* | | | | | | |

**Table S3** Logistic regression model of variables associated with dying in the first year of dialysis treatment versus surviving, in lower Body Mass Index patients (<23.1 kg/m^2^) measured at baseline.

| **Characteristics** | **Odds ratio (95% Confidence Interval)** | |
| --- | --- | --- |
|  | **Model 1** | **Model 2** |
| Primary kidney disease (%) |  | *N.A.* |
| Diabetes Mellitus | 1.00 (ref) |  |
| Glomerulonephritis | 0.13 (0.03 - 0.71)* |  |
| Renal vascular disease | 0.48 (0.19 - 1.21) |  |
| Other | 0.27 (0.11 - 0.66)** |  |
| Heart failure | 2.53 (1.38 - 4.63)** | 2.12 (1.06 - 4.27)* |
| Diabetes mellitus | 2.62 (1.32 - 5.18) ** | 2.37 (1.08 - 5.19)* |
| Peripheral vascular disease | 3.09 (1.72 - 5.55)*** | 3.95 (1.92 - 8.12)*** |
| Malignancy | 1.96 (1.02 - 3.77)* | 2.55 (1.19 - 5.49)* |
| Chronic lung disease | 2.40 (1.08-5.33)* |  |
| EQ5D mobility |  |  |
| no limitations in walking | 1.00 (ref) |  |
| some limitations in walking | 3.63 (1.29 - 10.2)* |  |
| confined to bed | 7.30 (1.89 - 28.2)** |  |
| Albumin (per 5g/L) | 0.75 (0.60 - 0.94)* | 0.73 (0.56 - 0.95)* |
| Urea (per 5 mmol/L) | 1.12 (1.01 - 1.25)* | 1.15 (1.01 - 1.30)* |
| *Model 1= univariable logistic regression; only the significantly associated characteristics are shown.*  *Model 2= all the characteristics of Model 1 were added into one multivariable logistic regression model, together with age and sex. Primary kidney disease was omitted of the model, because diabetes mellitus (which showed the highest risk of dying in the univariable model) was already included in the model.*  *N.A., not applicable. * P<0.05; ** P<0.005; ***P<0.001.* | | |

**Figure S1** Kaplan Meier survival curves for all-cause mortality by BMI tertiles.


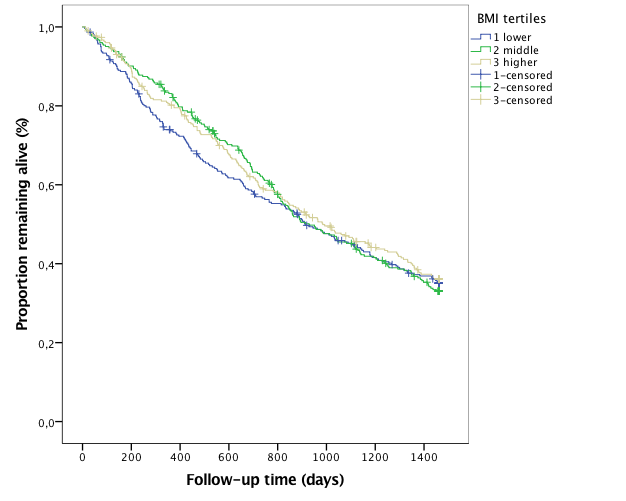


*Fig S1. Kaplan Meier survival curves for all-cause mortality by BMI tertiles in 908 elderly participants of the NECOSAD study from start of dialysis until 4 years of follow-up. Log-rank test P= 0.8.*
